# Magnetic Resonance Colonography May Predict the Need for Bowel Resection in Colorectal Endometriosis

**DOI:** 10.1155/2017/5981217

**Published:** 2017-09-25

**Authors:** Arnaldo Scardapane, Filomenamila Lorusso, Mariantonietta Francavilla, Stefano Bettocchi, Fabiana Divina Fascilla, Giuseppe Angelelli, Marco Scioscia

**Affiliations:** ^1^Interdisciplinary Department of Medicine, Section of Radiology, University of Bari Medical School, Bari, Italy; ^2^Department of Radiology, IRCCS “De Bellis” Hospital, Castellana Grotte, Italy; ^3^Department of General and Specialist Surgical Sciences, Section of Obstetrics and Gynecology, University of Bari Medical School, Bari, Italy; ^4^Department of Obstetrics and Gynecology, Sacro Cuore Don Calabria Hospital, Negrar, Verona, Italy

## Abstract

**Purpose:**

To define if MRI findings in patients with deep pelvic endometriosis (DPE) may be predictive for the need of bowel resection.

**Material and Methods:**

A retrospective survey of 196 pelvic MRIs of women who received laparoscopic procedures for DPE was carried out. A pelvic MRI was performed in all patients: it consisted in T2w-TSE sequences in axial, sagittal, and coronal planes and T1w and THRIVE sequences in the axial plane; the exam was completed by MR-Colonography. Intestinal lesions were measured in short and long axis and the degree of stenosis was established. A multivariate logistic regression was used to identify the predictors of intestinal resection.

**Results:**

57/196 patients received an intestinal resection. Multivariate logistic regression demonstrated a predictive value of short axis (Odds-Ratio = 2.29, *p* = 0.011) and stenosis (Odds-Ratio = 1.20, *p* = 0.003). ROC analysis showed that a cut-off value of 11 mm for the short axis and 30% for the stenosis may correctly classify, respectively, 96,94% (sensitivity 92,9% and specificity 98,56%) and 97,96% (sensitivity 94,74% and specificity 99,3%) of the cases.

**Conclusion:**

The presence of an endometriotic rectal nodule > 11 mm in short axis causing a stenosis > 30% in pelvic MRI reliably predicts the need of a rectal resection.

## 1. Introduction

Deep pelvic endometriosis (DPE) is defined by the presence of lesions penetrating the retroperitoneal space or the wall of pelvic organs to a depth of at least 5 mm and resulting in fibrosis and muscular hyperplasia [1]. Intestinal endometriotic involvement occurs in 4–37% of patients with DPE [2]. The rectosigmoid colon is the most frequent bowel location, accounting for 85% of all endometriotic bowel lesions [3, 4]; in addition to the rectosigmoid junction, the most common intestinal segments affected are, in descending order of frequency, the appendix (2–18%), the distal ileum (2–16%), and the caecum (<2%) [5]. Intestinal endometriosis can cause severe symptoms such as diarrhea, dyschezia, bowel cramping, and pain on defecation, which can negatively affect the quality of life and may progress notwithstanding medical therapy [6, 7]. Many studies have demonstrated that laparoscopy is a safe option in the treatment of bowel endometriosis [8, 9]; anyway, laparoscopic colorectal resection generally exceeds the competence of gynecologists and should be performed by a multidisciplinary surgical team (general surgeon with gynecologist). To ensure an adequate surgical approach in these cases, it is necessary to provide a complete depiction of all pelvic lesions, and colorectal ones in particular, in order to identify women needing a segmental resection [10]. This represents a fundamental aspect both in informed consent and in planning a multidisciplinary approach. Transvaginal, transrectal, and endoscopic transrectal ultrasounds have demonstrated high accuracy in recognizing intestinal involvement, and their capability in assessing the penetration of rectal nodules is promising [11–14]. Although endometriosis localization occurs more frequently in the proximal and medium part of the rectum, it is not infrequent that endometriosis occurs in the distal and medium part of the sigmoid colon that transrectal and transvaginal ultrasound cannot assess. In some cases, echoendoscope can be used but it is unlikely to perform this exam in all patients with endometriosis unless a MR or CT imaging suggests proximal bowel endometriosis. Furthermore, ultrasonography can easily evaluate the infiltration and the extension of the disease while the stenosis is detected less by ultrasound than by imaging studies [15]. Nowadays, pelvic MRI is first-line exam for detection of deep infiltrating endometriosis and for colorectal involvement in symptomatic patients albeit its power to predict, on the basis of imaging findings, whether a bowel resection will be necessary or not is still unclear.

The aim of the present study is to establish if pelvic MR-findings and the estimation of the degree of colorectal stenosis in bowel endometriosis may play a predictive role in surgery for a radical treatment in women with DPE. For this purpose, we have proposed a new formula for the measurement of the degree of stenosis based on MR-Colonographic images.

## 2. Materials and Methods

After obtaining the authorization from our Institutional Review Boards, we retrospectively identified 196 women who received an operative laparoscopy for DPE and a pelvic MRI between October 2012 and December 2015 (mean age 35.35 ± 6.7).

The need for laparoscopic surgery was established by the gynecological team on the basis of clinical findings with the following indications: (1) DPE with suspicious colorectal involvement, accompanied by severe pelvic pain (dysmenorrhea, dyschezia, dyspareunia, nonmenstrual pelvic pain, and intestinal cramping) resistant to medical treatment; (2) symptomatic or asymptomatic severe DPE in women suffering from infertility for at least 1 year. The MR examination was performed in all surgical candidates to obtain an accurate evaluation of pelvic disease.

After obtaining a written informed consent, including the possibility of a bowel resection, all surgical procedures were performed by the same surgical team (including two gynecologists and one general surgeon in all the cases) with an interval between MRI and surgery ranging from 4 to 46 weeks (mean 18 weeks).

Since endometriosis may determine important adhesion of the posterior pelvic floor compartment, and this may confuse preoperative and intraoperative images, the decision whether to perform bowel surgery is usually made during surgery after adhesiolysis. Therefore, according to intraoperative findings, the general surgeon decided the type of surgical approach (bowel shaving or segmental resection) or doing nothing if the bowel appeared not stenotic. In fact, several authors have supported a less aggressive management of endometriosis of the bowel in favor of a superficial removal of the nodule (shaving technique) or just detaching the bowel from the rectovaginal septum when the nodule appears not stenotic [16, 17].

Major surgery such as colorectal segmental resection with terminoterminal anastomosis was carried out in 57/196 (29%) patients, while conservative treatment such as bowel shaving (excision of serosal and muscular layers infiltrated by endometriosis without opening the mucosal layer) or adhesiolysis was performed in 139/196 (71%) women.

All histological findings were examined in order to confirm the presence of endometrial tissue, with particular concern to intestinal infiltration.

Preoperative workup included bimanual palpation, assessment of Cancer Antigen 125 levels, vaginal and abdominal ultrasonography, and standard pelvic MRI, completed by MR-Colonography to visualize the whole large bowel.

### 2.1. MRI Technique and Image Interpretation

MRI examinations were performed in all cases with a 1.5-T scanner (Philips, Achieva 1.5) using a 4-channel surface coil in 102/196 (52%) and a 16-channel surface coil in 94/196 (48%) patients, regardless of the phase of the menstrual cycle, according to the protocol consisting of a Standard High Resolution pelvic MRI followed by a MR-Colonography (as previously described in literature) [18]. On the day before the investigation, all patients underwent bowel preparation with administration of 2 doses of PEG 4000 granular powder (SELGE 1000, Promefarm, Italy) dissolved in 1000 ml of water per dose. Patients were asked to refrain from voiding for 30 minutes before the procedure, and an antiperistaltic drug (10 mg butylscopolamine (Buscopan), Boehringer Ingelheim, Germany) was injected intramuscularly just before imaging.

Standard high resolution pelvic MRI consisted of the following sequences:

- T2-weighted Turbo Spin Echo (TSE) in axial, coronal, and sagittal planes (matrix 384 × 512; Field of View (FOV) 260; Number of Signal Averages (NSA) 3; TE 110 msec; shortest TR; section thickness 5-6 mm; 24 sections; acquisition time 3 min and 30 s)
- T1-weighted TSE in coronal and sagittal planes (matrix 512 × 512; FOV 260; NSA 2; TE 110 msec; shortest TR; section thickness 5-6 mm; 24 sections)
- T1-weighted High-Resolution-Isotropic-Volume-Excitation (THRIVE) sequences in the axial plane (matrix 256 × 256; FOV 350; NSA 2; shortest TE/TR; section thickness 2 mm; 80 slices).

MR-Colonography consisted of the following sequences acquired once a complete colonic distension was achieved by administering 1.5–2 L of water via rectal tube:

- Balanced Turbo Filed Echo (BTFE) sequences in axial, coronal, and sagittal planes (matrix 256 × 256; FOV 350–450; shortest TE/TR; section thickness 5 mm; 40 sections; breath-hold acquisition).
- Single-Shot Fast Spin Echo (SS FSE) T2-weighted (T2W) sequences in axial, coronal, and sagittal planes (thickness 4-5 mm; TE 100 ms; shortest TR; flip angle 90°; matrix 320 × 320; FOV 350–380 mm; breath-hold acquisition)

**Figure 1:**
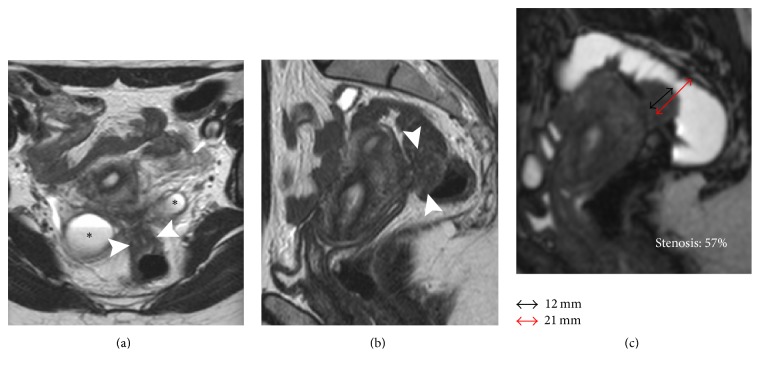
32-year-old patient with DPE and infiltrative nodule requiring segmental resection. (a) Axial T2W image, (b) sagittal T2W image, and (c) MR-Colonography. Pelvic tethering involving the ovaries and the rectum with Douglas pouch obliteration. An infiltrative nodule (short axis 12 mm) is visible on the anterior wall of the rectum (arrowheads). Presence of bilateral endometriomas (*∗*). MR-Colonography demonstrates a stenosis of 57% (c).

All MR images were reviewed in consensus by two senior radiologists (FL and AS with 6 and 14 years of experience, resp.). The diagnosis of DPE and its bowel involvement was established on standard pelvic MR images according to the criteria suggested by Bazot et al. [19, 20] on the basis of the presence of morphological abnormalities consisting in low signal intensity nodules or spiculated masses on T2-weighted sequences, associated with high signal intensity spots corresponding to hemorrhagic foci on T1-weighted and/or fat-suppressed sequences. The following endometriotic lesions were recorded: endometriomas, adenomyosis, and the involvement of the torus uterinus, the uterosacral ligaments (USL), the rectovaginal septum (RVS), and the bowel. In particular, for each bowel lesion, the long and short axes were measured, while the degree of stenosis was determined using MR-Colonographic images through this formula: degree of stenosis (%) = protruding nodule short axis/colonic diameter × 100 (Figure 1). The finding of the mushroom-cap sign (MCS), formerly described by Yoon et al., was also annotated [21]. Tethering of pelvic structures and loss of the corresponding cleavage planes, without appreciable nodular lesions, suggested a diagnosis of Douglas pouch obliteration caused by adhesion.

### 2.2. Statistical Analysis

On the basis of pelvic MRI, four numeric variables (age, long and short axis diameters, and degree of stenosis) and 9 dichotomous variables (presence/absence of the following MR signs: recognizable bowel nodules, MCS, pelvic tethering, Douglas pouch obliteration, SRV, Torus, USL involvement, adenomyosis, and endometriomas) were categorized.

Firstly, we performed a bivariate logistic regression between the outcome variable “resection” and the other variables; all significant variables of this first model (*p* < 0.05) were considered as potential predictors and employed to run a stepwise logistic regression. The values of *p* < 0.05 and *p* > 0.1 were used, respectively, to enter and to remove a variable from the model. The diagnostic performance of these predictors was, finally, investigated by Receiver Operating Characteristic (ROC) analysis. Statistical analysis was performed by the software STATA version 14.

## 3. Results

All the women completed the MR evaluation without complications and images were considered adequate for diagnosis in all cases. Detailed results of MR evaluation of 196 patients are listed in Tables 1 and 2. Intestinal lesions were identified in 82/196 (41,8%) patients by MRI and in 83/196 (42,3%) cases during surgery (sensitivity 96%, specificity 98%, NPP 97%, PPV 98%, and accuracy 97%). In three cases only both MRI and surgery identified two colorectal nodules in the same patient. MRI images showed lesion sizes from 5 mm to 25 mm (mean 13 mm) in short axis and from 5 mm to 55 mm (mean 23,6 mm) in long axis, whereas the estimated degree of stenosis ranged from 0% to 80% (mean 33%).

**Figure 2:**
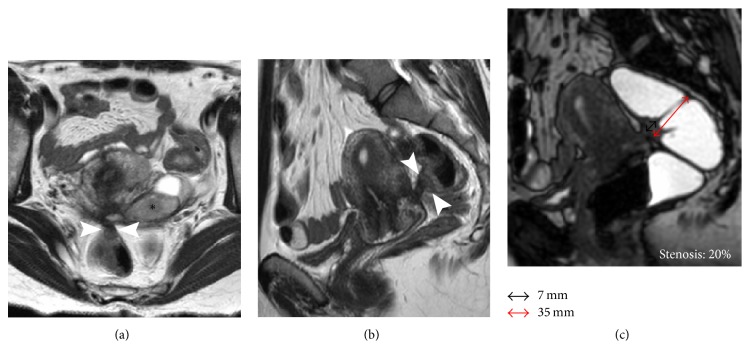
29-year-old woman with DPE and infiltrative nodule requiring conservative serosal shaving of the rectum. (a) Axial T2W image, (b) sagittal T2W image, and (c) MR-Colonography. Pelvic tethering involving the left ovary and the rectum with Douglas pouch obliteration. A small infiltrative nodule (short axis 7 mm) is visible on the anterior wall of the rectum (arrowheads). Presence of left endometrioma (*∗*). MR-Colonography demonstrates a low degree of stenosis of 20% (c).

57/196 (28,8%) patients received a bowel resection (rectum *n* = 51; sigmoid *n* = 5; cecum *n* = 1) (Figure 1), while a conservative treatment was performed in 139/196 (71%) women; among them 26/196 (13%) with recognizable bowel nodules (rectum *n* = 24 and sigmoid *n* = 2) received a bowel shaving (Figure 2). Histopathology confirmed endometriosis tissue in all specimens. The results of bivariate regression analysis are shown in Table 3. In this first analysis the absence of a bowel nodule at MRI behaved as perfect predictor for a conservative treatment leading to the removal of the corresponding variable from the model. All remaining variables with a significant correlation with the outcome variable (resection) were used to run a stepwise logistic regression which demonstrated that only the short axis (Odds Ratio = 2.29 (1.205–4.35); *p* = 0.011) and the degree of stenosis (Odds Ratio = 1.20 (1.066–1.35); *p* = 0.003) play a significant role as predictors of the final outcome (Table 4). In addition to this, ROC analysis demonstrated that a cut-off value of 11 mm for the short axis and 30% for the degree of stenosis, as predictors, may correctly classify, respectively, 96,94% (sensitivity 92,9%, specificity 98,56%, and area under the curve (AUC) 0.993) and 97,96% (sensitivity 94,74%, specificity 99,3%, and AUC 0,989) of the cases (Table 5).

## 4. Discussion

In the evaluation of colorectal endometriosis, the estimation of luminal stenosis is crucial as it drives the surgeon to make a decision during surgery whether to resect the bowel although at present there is no consensus about an imaging parameter that may foresee this need [22]. In this retrospective study, we described a new method for the measurement of endometriotic rectosigmoid stenosis to investigate the ability of pelvic MRI to predict the need for bowel resection in women with DPE. Namely, our analysis demonstrated

- a very high accuracy of MRI in recognizing colorectal involvement and showed that an intestinal resection is very unlikely in patients without detectable bowel nodules at pelvic MRI. In our series we found 2 false negative and 3 false positive cases, even though none of these women underwent a bowel resection because of the small size of nodules;
- that colorectal lesions are usually associated with severe deep endometriosis involving other pelvic structures such as RVS, USL, and Douglas pouch, while ovarian and uterine localizations as endometriomas and adenomyosis without recognizable DPE lesions showed no correlation with intestinal involvement;
- that the degree of stenosis as measured on MR-Colonographic images plays a predictive role in surgical treatment.

These findings, which seem somehow obvious, are useful, in our view, as they suggest that radiologists should perform a careful search for colorectal lesions in women with evidence of severe deep endometriosis, and this leads to an increase in the MRI accuracy that is still considered low for less experienced radiologists [23]. The crucial finding of this study concerns the possibility of predicting an intestinal resection by measuring the short axis of the nodule and the degree of luminal stenosis. In other words, using a cut-off value of 11 mm for the short axis and 30% for the stenosis, according to ROC analysis, we could correctly identify, respectively, 96,94% and 97,96% of patients undergoing bowel resection. This information, before laparoscopy, is fundamental to choose the best surgical approach (nodulectomy versus bowel resection) with the colorectal surgeons and to obtain informed consent from the woman [23–25]. According to the American Fertility Society, MRI has proved to be more accurate than laparoscopy in detection of all deep sites of endometriosis for a complete map before surgery [26, 27], although the capabilities of this exam in predicting intestinal resection are still unclear. Many authors tried to assess the degree of wall infiltration by US, MRI, or CT correlating histopathology to imaging finding [21, 24]. Both transvaginal and transrectal sonography demonstrated a good capability in estimation of bowel infiltration; however, their field of view is limited: in fact, consistent results were achieved in investigation of rectum, while proximal bowel can be only visualized using flexible echoendoscopes which are not routinely used in first level centers [28–30]. In our opinion, CT and MRI have too low resolution to assess the depth of bowel infiltration and this limitation is widely demonstrated in literature. In fact, it excludes, regarding the staging of rectal cancer, the possibility that MRI (or CT) can detect neoplastic infiltration of each layer of the bowel wall which, on the contrary, is better visualized by transrectal US [31, 32]. Moreover the exact evaluation of the parietal layer reached by the disease has no prognostic value but it helps to predict the need for bowel resection. For these reasons, we did not try to correlate our findings with histopathology, but only with surgery that has impact directly on the outcome. Our results show that a preoperative morphologic evaluation of the nodule, by measuring its short axis and the degree of luminal stenosis, answers with high degree of accuracy to the main question of the surgeon, whether an intestinal resection will be required or not. Our findings are in line with large surgical series where colorectal resections were demanded in nodules > 2 cm with a degree of stenosis > 50% [33, 34]. However, predictive cut-off values that we found in our series seem to be smaller (11 mm and 30%) than those expected by bowel surgeons. According to our view, this is only an apparent discrepancy: surgeons often use the long axis to define nodular size while our cut-off value of 11 mm is related to the short axis; in addition, the degree of stenosis in our series was calculated on MR-Colonography after water distension of the rectal lumen, and it is likely to be lower than the one that appears at surgery with collapsed loops.

This study suffers from the limitation of a retrospective assessment and our results should be confirmed by a prospective study. In addition, a correct estimate of the stenosis needs the standard pelvic MR to be completed with the MR-Colonography, which makes the exam more invasive and longer. However, as the MR staff of our institution is used to this examination and the protocol of MR-Colonography consists in breath-hold fast sequences, the additional exam time is limited to 5 to 10 minutes with a minimum discomfort for the patients.

In conclusion, our results highlight a very important role for MRI in predicting intestinal resection in patients with colorectal endometriosis and suggest to radiologists the need of a systematic mention in their report of the short axis and the degree of stenosis related to endometriotic bowel nodules. This information helps surgeons to schedule the operation time correctly and predict the need of a multidisciplinary approach (gynecologist and general surgeon) and of a specific informed consent. However, our results rely on a retrospective study and need further investigation to confirm the role of MRI and of the proposed formula for the measurement of intestinal stenosis on a prospectively enrolled series.

## Figures and Tables

**Table 1:** Endometriotic pelvic lesion at MRI.

Lesion	Cases/total	%
Endometriomas	156/196	79,6%
Adenomyosis	76/196	38,8%
Rectovaginal septum	95/196	48,5%
Uterosacral ligaments	64/196	32,7%
Torus	107/196	54,6%
Pelvic tethering	120/196	61,2%
Douglas obliteration	126/196	64,3%

**Table 2:** Endometriotic bowel nodules identified at MRI and Surgery.

Bowel nodules			
	Surgery +	Surgery −	
MRI +	80	2	82
MRI −	3	111	114

	83	113	196

Sensitivity 96%, specificity 98%, PPV 98%, NPV 96%, and accuracy 96%.

**Table 3:** Bivariate relationship between the outcome “resection” and MR-findings of deep endometriosis.

	Coef.	Std. Err.	[95% Conf. interval]		*p*
Bowel nodule	Removed from the model as the absence of a nodule perfectly predicts the outcome “no resection”				
AGE	0.0554707	0.0241343	.0081683	0.102773	0.022^**∗**^
Endometriomas	0.0975612	0.3952	−0.6771128	0.8722353	0.805
Adenomyosis	0.5026289	0.3192016	−0.1229947	1.128252	0.115
RVS	0.9491915	0.3265181	0.3092278	1.589155	0.004^**∗**^
Torus	1.041788	0.3402064	0.3749958	1.70858	0.002^**∗**^
USL	1.453953	0.3344335	0.7984753	2.109431	0.000^**∗**^
Tethering	1.301068	0.3762265	0.563678	2.038459	0.001^**∗**^
Douglas	3.271014	0.7395757	1.821472	4.720556	0.000^**∗**^
Short axis	0.9745481	0.2324707	0.5189139	1.430182	0.000^**∗**^
Long axis	0.3578067	0.0674774	0.2255534	0.4900601	0.000^**∗**^
Stenosis	0.2630366	0.0494875	0.1660429	0.3600302	0.000^**∗**^
MCS	5.331359	0.6346775	4.087414	6.575304	0.000^**∗**^

**Table 4:** Predictors of the outcome “resection” identified according to stepwise logistic regression.

	Odds Ratio	Std. Err.	*z*	*p*	[95% Conf. interval]	
Short axis	2.292253	.7513042	2.53	0.011^*∗*^	1.205798	4.357633
Stenosis	1.200366	.0726209	3.02	0.003^*∗*^	1.066146	1.351483
_cons	.0000152	.0000551	−3.06	0.002	1.25*e* − 08	.0184717

**Table 5:** ROC tab analysis evaluating the performance of the variables “short axis” and “stenosis” in predicting the need of intestinal resection.

	Cut off	Correctly classified	Sensitivity	Specificity	AUC	Standard error	Confidence interval
Short axis	≥11 mm	96,94%	92,9%	98,56%	0,993	0,0036	0,98–1
Stenosis	≥30%	97,96%	94,74%	99,3%	0,989	0,009	0,97–1
